# Supplementary material for: Microbial risk score for capturing microbial characteristics, integrating multi-omics data, and predicting disease risk
Source: Microbiome. 2022 Aug 5;10:121. doi: 10.1186/s40168-022-01310-2 (PMC9354433; doi:10.1186/s40168-022-01310-2)
Supplement: Supplementary file 2 — Additional file 1: Figure S1. The ROC curves and AUC values for various ML algorithms to predict the alive or deceased status in the NYULH COVID-19 cohort. A. Predication performance for elastic-net logistic regression (glmnet), penalized discriminant analysis (pda2), regularized random forest (RRF), and neural networks with feature extraction (pcaNNet) methods. B. Predication performance for naive Bayes (naïve_bayes), neural network (nnet), stochastic gradient boosting (gbm), and support vector machines with polynomial kernel (svmPoly) methods. Figure S2. The AUC values and 95% CIs for MRSαs to classify healthy and nonhealthy and two disease conditions in the discovery and validation GMHI cohorts [27], respectively. CA: colorectal adenoma, CC: colorectal cancer, CD: Crohn’s disease, and RA: rheumatoid arthritis. Figure S3. Heatmaps of Spearman’s rank correlations between the top 50 taxa from metagenome and the top 50 taxa from metatranscriptiome, in the alive and deceased groups, separately. The top 50 features were selected based on the proportion of selection in all CV iterations. Figure S4. Heatmaps of Spearman’s rank correlations between the top 50 taxa from metatranscriptome and the top 50 genes from host transcriptome, in the alive and deceased groups, separately. The top 50 features were selected based on the proportion of selectin in all CV iterations. Figure S5. Comparisons among various MRSs in terms of AUC value and 95% CI in the discovery and validation cohorts [27]. Here candidate taxa are identified by ANCOM-BC [31], ALDEx2 [56], and Maaslin2 [57], and the MRSαs are constructed by Shannon, Simpson, and Observed indices, respectively. DA: differential abundance, CA: colorectal adenoma, CC: colorectal cancer, CD: Crohn’s disease, and RA: rheumatoid arthritis. Figure S6. The mean and standard derivation of the ranks of MRSα’s AUCs with ANCOM-BC, ALDEx2, and Maaslin2, respectively. For each alpha diversity index in each comparison of two diseases or healt [file 40168_2022_1310_MOESM1_ESM.docx]

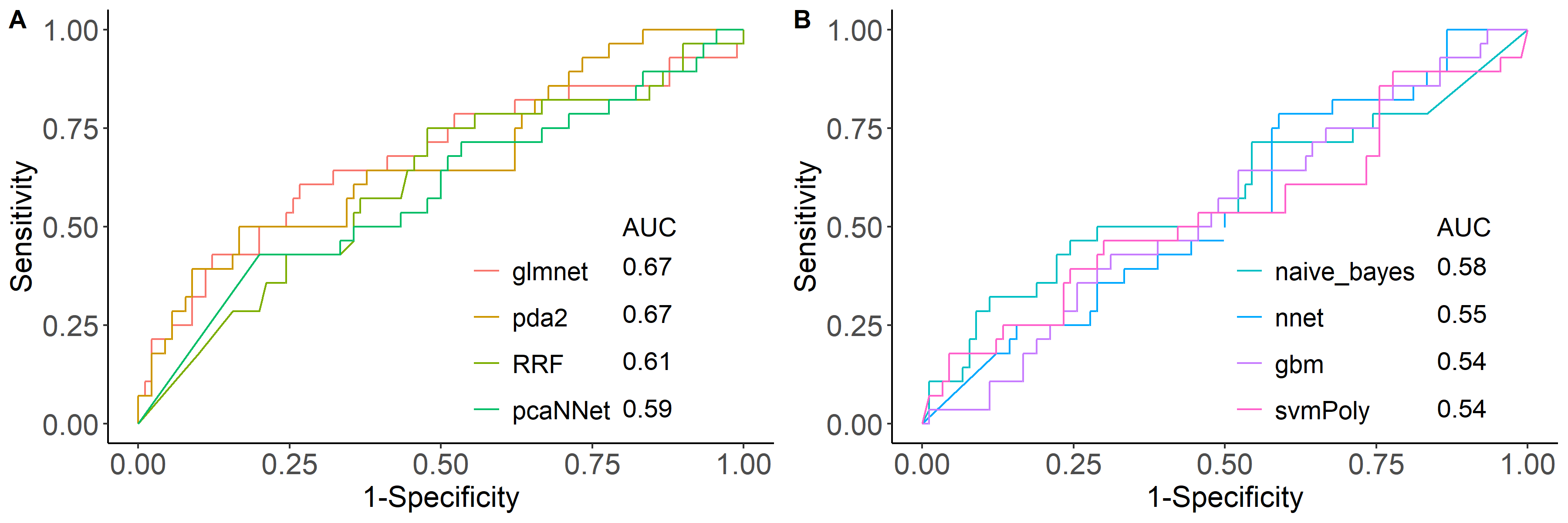


**Figure S1.** The ROC curves and AUC values for various ML algorithms to predict the alive or deceased status in the NYULH COVID-19 cohort. A. Predication performance for elastic-net logistic regression (glmnet), penalized discriminant analysis (pda2), regularized random forest (RRF), and neural networks with feature extraction (pcaNNet) methods. B. Predication performance for naive Bayes (naïve_bayes), neural network (nnet), stochastic gradient boosting (gbm), and support vector machines with polynomial kernel (svmPoly) methods.


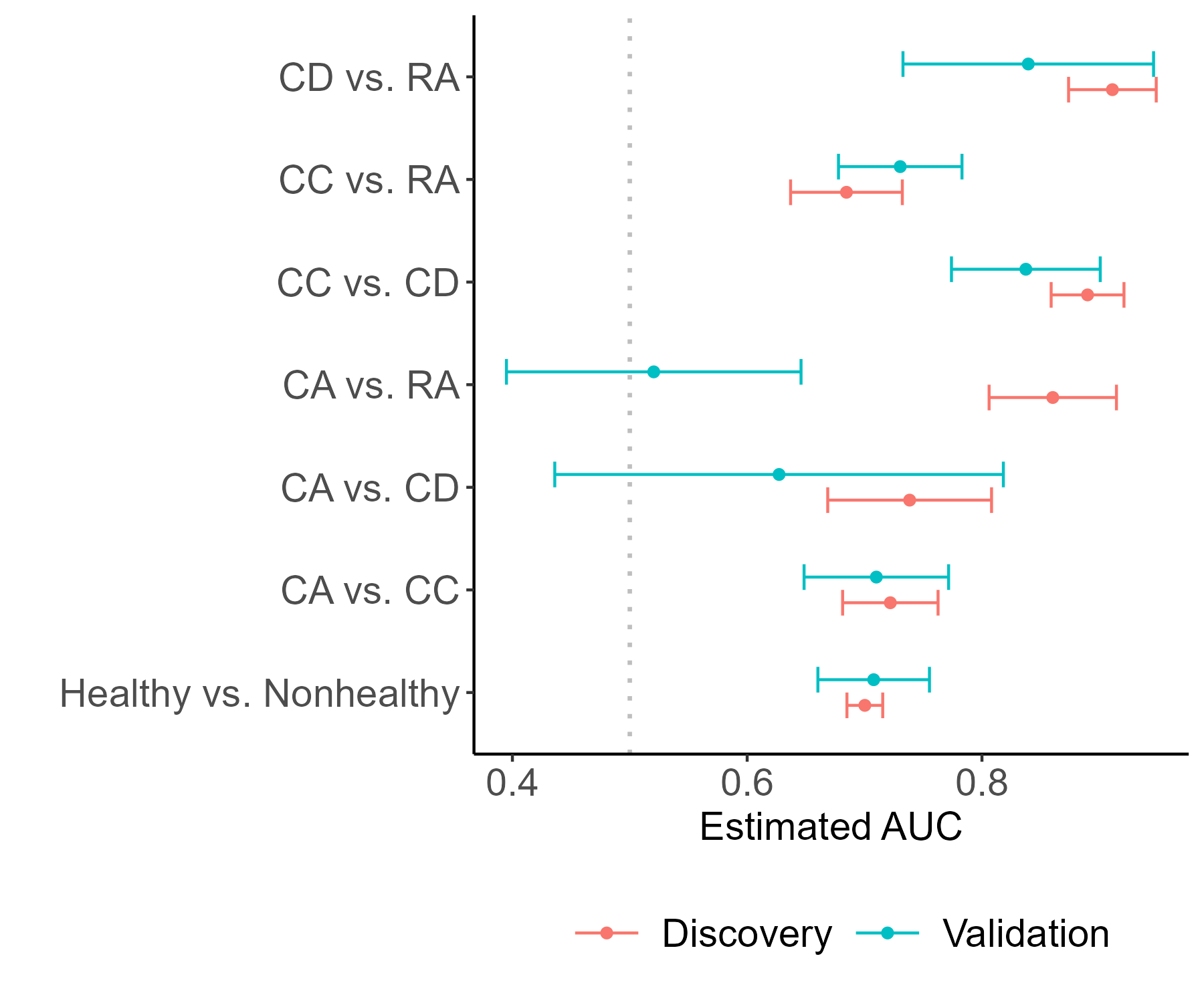


**Figure S2.** The AUC values and 95% CIs for $\mathrm{MRS}_{\alpha}$s to classify healthy and nonhealthy and two disease conditions in the discovery and validation GMHI cohorts [1], respectively. CA: colorectal adenoma, CC: colorectal cancer, CD: Crohn’s disease, and RA: rheumatoid arthritis.


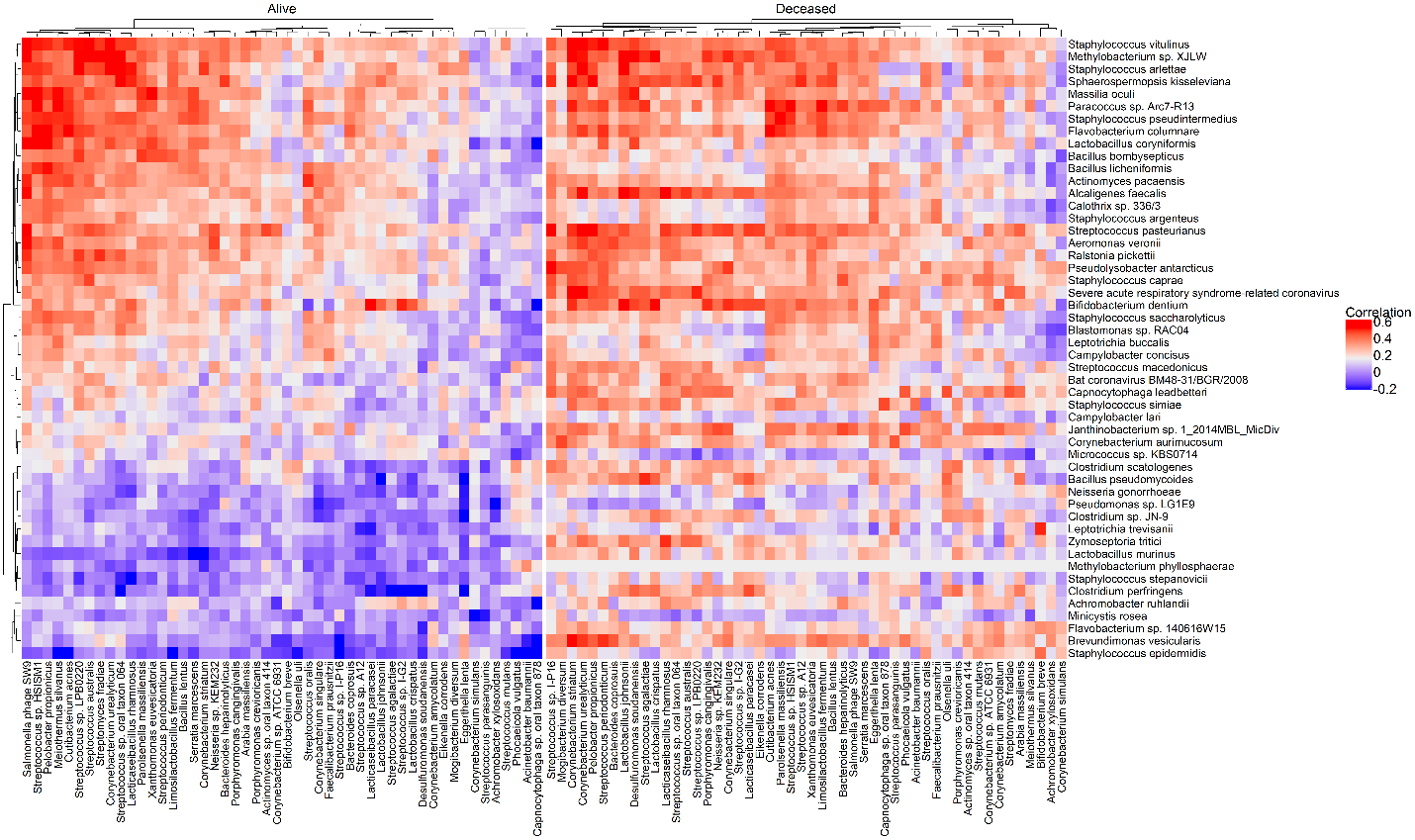


**Figure S3.** Heatmaps of Spearman’s rank correlations between the top 50 taxa from metagenome and the top 50 taxa from metatranscriptiome, in the alive and deceased groups, separately. The top 50 features were selected based on the proportion of selection in all CV iterations.


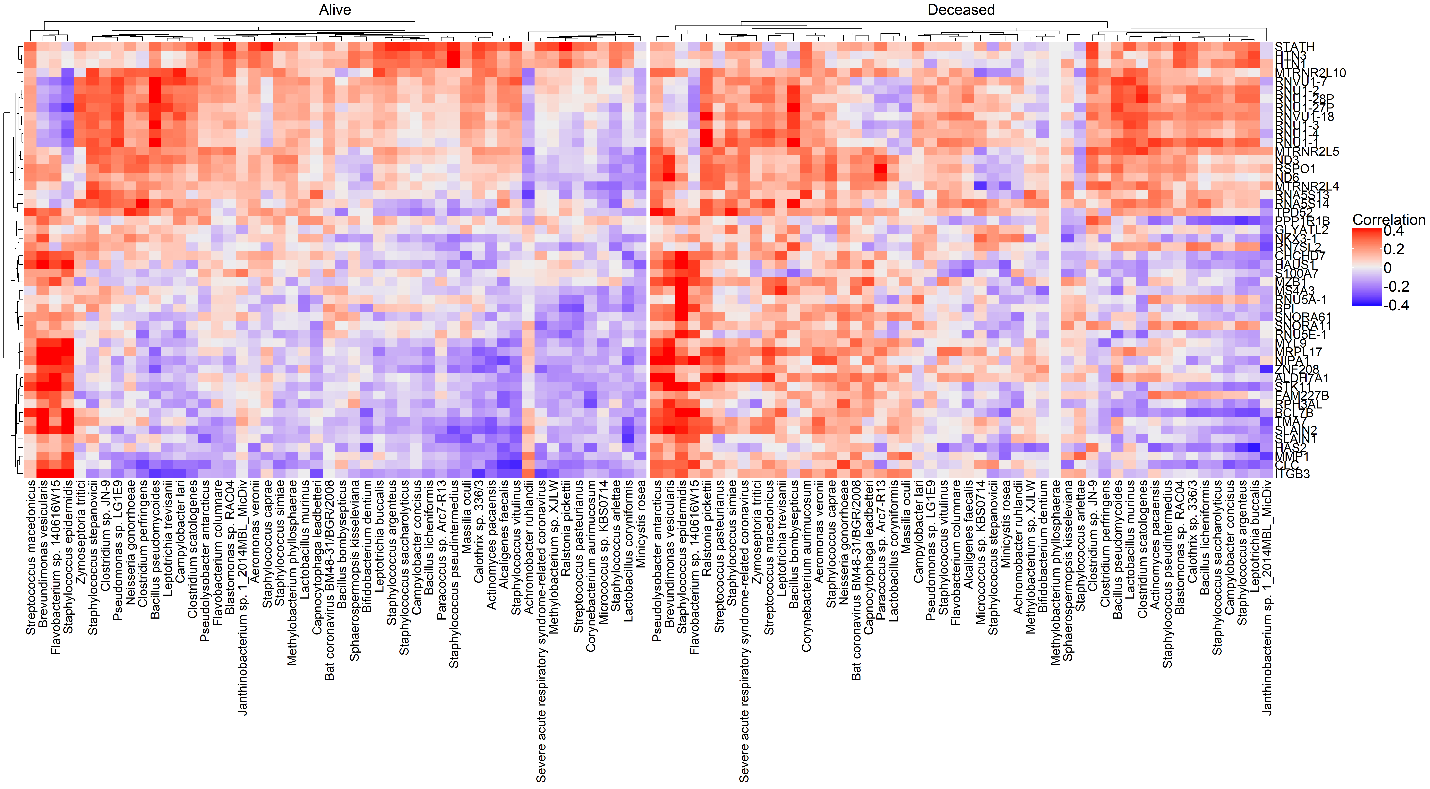


**Figure S4.** Heatmaps of Spearman’s rank correlations between the top 50 taxa from metatranscriptome and the top 50 genes from host transcriptome, in the alive and deceased groups, separately. The top 50 features were selected based on the proportion of selectin in all CV iterations.


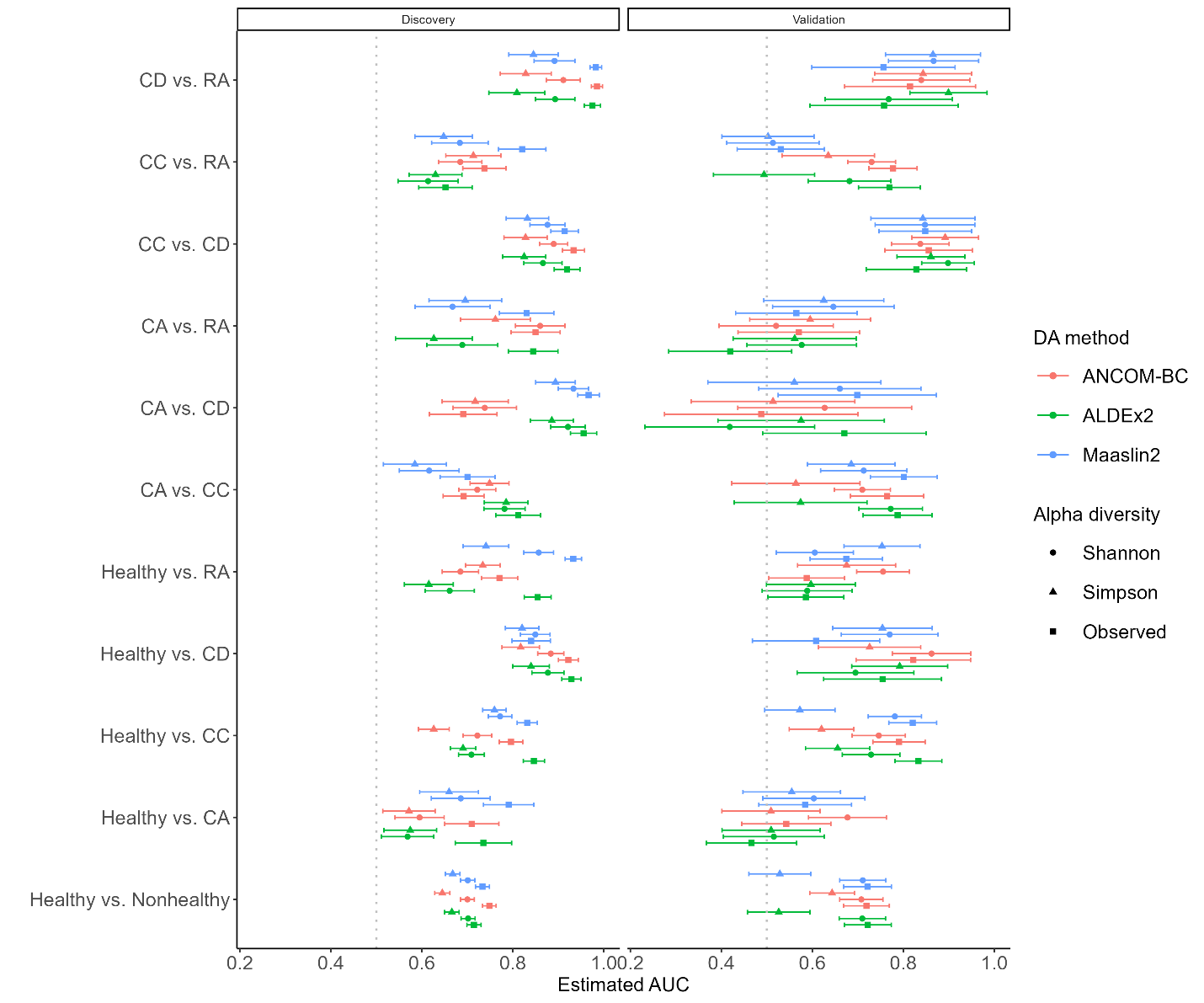


**Figure S5.** Comparisons among various MRSs in terms of AUC value and 95% CI in the discovery and validation cohorts [1]. Here candidate taxa are identified by ANCOM-BC [2], ALDEx2 [3], and Maaslin2 [4], and the $\mathrm{MRS}_{\alpha}$s are constructed by Shannon, Simpson, and Observed indices, respectively. DA: differential abundance, CA: colorectal adenoma, CC: colorectal cancer, CD: Crohn’s disease, and RA: rheumatoid arthritis.


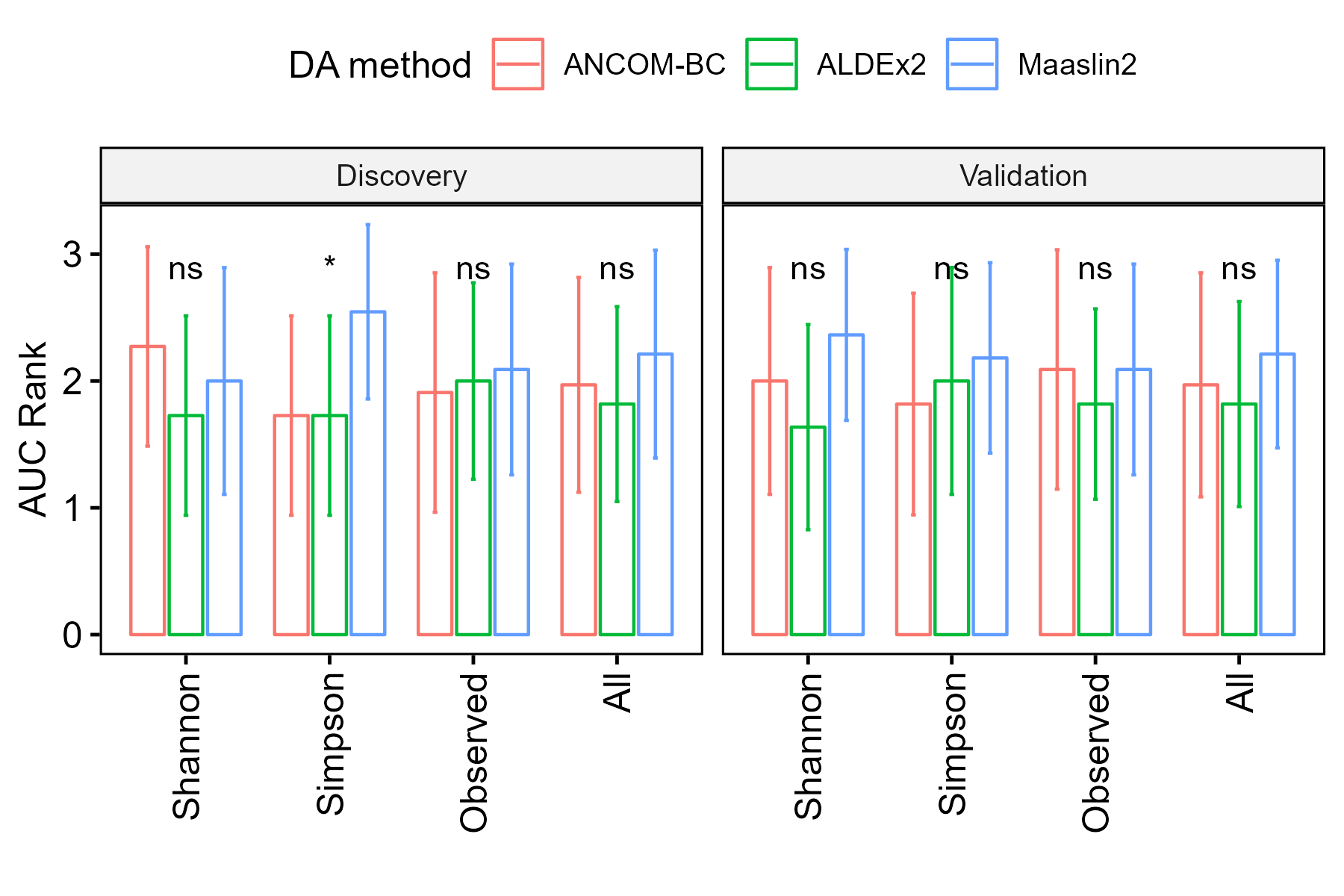


**Figure S6**. The mean and standard derivation of the ranks of $\mathrm{MRS}_{\alpha}$’s AUCs with ANCOM-BC, ALDEx2, and Maaslin2, respectively. For each alpha diversity index in each comparison of two diseases or healthy conditions, the AUCs of $\mathrm{MRS}_{\alpha}$ with three DA methods were ranked 1-3. A higher rank represents a higher AUC. For each alpha diversity index, the Kruskal-Wallis test was performed to check difference among three DA methods. All: all samples were used for test. Statistical significance: ns: *p*-value>0.05; *: *p*-value$\leq$0.05.


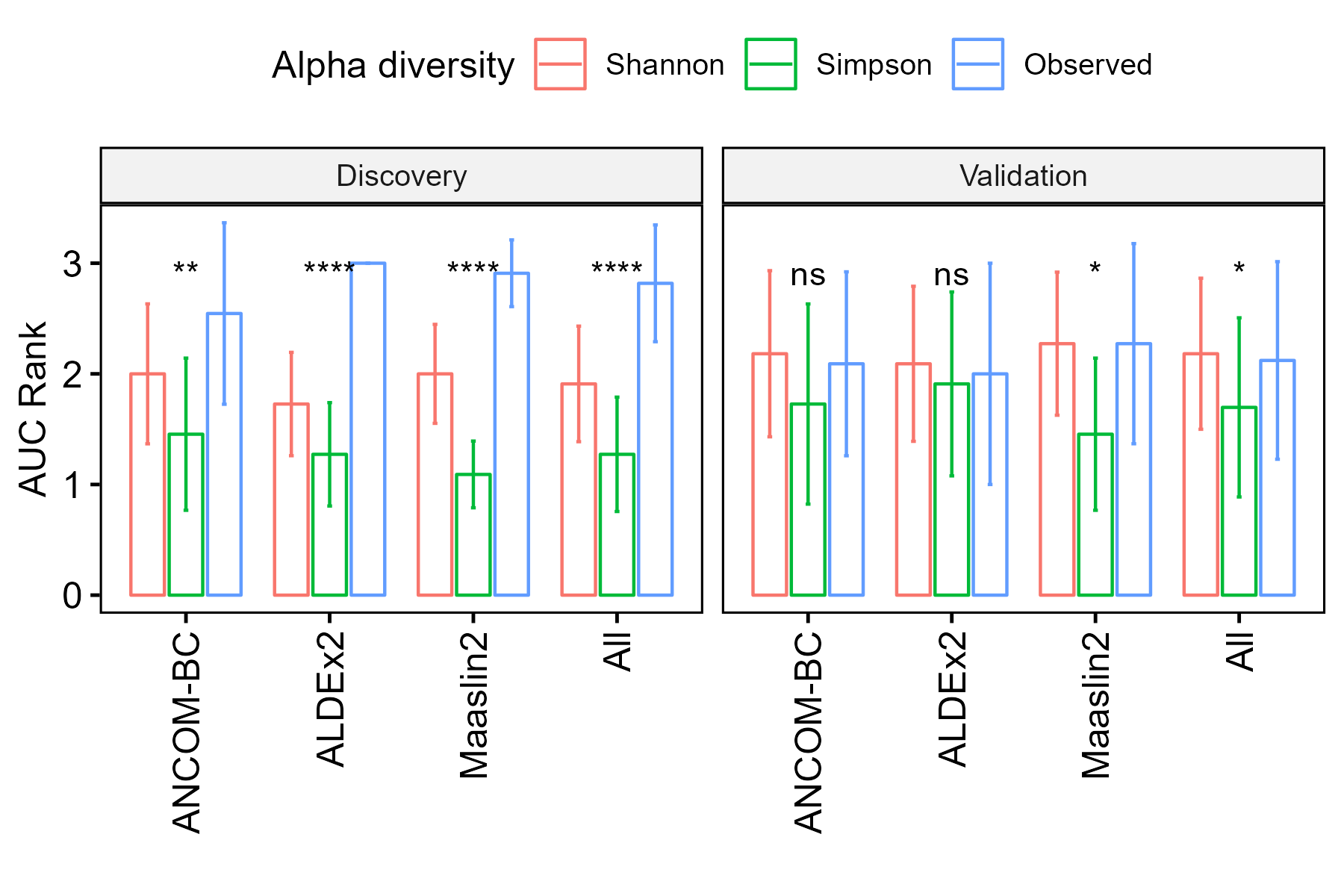


**Figure S7**. The mean and standard derivation of the ranks of $\mathrm{MRS}_{\alpha}$’s AUCs with Shannon, Simpson, and Observed indices, respectively. For each DA method in each comparison of two diseases or healthy conditions, the AUCs of $\mathrm{MRS}_{\alpha}$ with three indices were ranked 1-3. A higher rank represents a higher AUC. For each DA method, the Kruskal-Wallis test was performed to check difference among three alpha diversity indices. All: all samples were used for test. Statistical significance: ns: *p*-value >0.05; *: *p*-value $\leq$0.05; **: *p*-value $\leq$0.01; ***: *p*-value $\leq$0.001; ****: *p*-value $\leq$0.0001.

**Reference**

1. Gupta VK, Kim M, Bakshi U, Cunningham KY, Davis JM, Lazaridis KN, Nelson H, Chia N, Sung J: **A predictive index for health status using species-level gut microbiome profiling**. *Nature communications* 2020, **11**(1):1-16.

2. Lin H, Peddada SD: **Analysis of compositions of microbiomes with bias correction**. *Nature communications* 2020, **11**(1):1-11.

3. Gloor G: **ALDEx2: ANOVA-Like Differential Expression tool for compositional data**. *ALDEX manual modular* 2015, **20**:1-11.

4. Mallick H, Rahnavard A, McIver LJ, Ma S, Zhang Y, Nguyen LH, Tickle TL, Weingart G, Ren B, Schwager EH: **Multivariable association discovery in population-scale meta-omics studies**. *PLoS computational biology* 2021, **17**(11):e1009442.
