# Supplementary material for: Microbial risk score for capturing microbial characteristics, integrating multi-omics data, and predicting disease risk
Source: Microbiome. 2022 Aug 5;10:121. doi: 10.1186/s40168-022-01310-2 (PMC9354433; doi:10.1186/s40168-022-01310-2)
Supplement: Supplementary file 4 — Additional file 3: Section S1. Computational details for risk scores. [file 40168_2022_1310_MOESM3_ESM.docx]

# **Section S1 Computational details for risk scores**

## $\mathbf{MR}\mathbf{S}_{\boldsymbol{\alpha}}$ calculation

Suppose the discovery cohort has $n$ subjects, $Q$ taxa and subscripts $i$, and $j$ indicate a subject and a taxon, respectively. For the $i$th subject, let $M_{ij}$ be the microbiome relative abundance with the constraint $\sum_{ij=1}^{Q} M_{ij}=1$. We perform ANCOM-BC [1] to test whether taxa are differentially abundant between cases and controls. Let $p_{j}$ be the p-value of the test results on the $j$th taxon correspondingly.

Here we consider $K$ p-value thresholds. Given a cut-off, the taxa with p-values less than the cut-off are selected and defined as a candidate sub-community. Specifically, for the $k$th p-value threshold, $Q^{k}$ taxa are selected and their relative abundances are $M_{ij_{k}}$, $j_{k}=1,\ldots,Q^{k},$ $k=1,\ldots,K$. The $\mathrm{MR}S_{\alpha}$ is calculated as:

$MRS_{\alpha_{k}}^{i}=\sum_{j=1}^{Q^{k}} \tilde{M}_{ij_{k}}\ln(\tilde{M}_{ij_{k}})$,

where $\tilde{M}_{ij_{k}}$ is relative abundance of the $j$th identified candidate taxon within the sub-community for the $i$th sample ($\tilde{M}_{ij_{k}}=\frac{M_{ij}}{\Sigma_{j=1}^{Q^{k}}M_{ij}}$). The AUC thereby is calculated and denoted as $\mathrm{AU}C_{k}$. The optimal p-value cutoff is defined as the value that produces the largest AUC among $K$ p-value thresholds,

$\hat{k}=\text{arg }\text{max}_{k}\left\{ \mathrm{AU}C_{k} \right\}_{k=1}^{K}$.

Given the optimal p-value cutoff $p_{\hat{k}}$, the associated taxa used for $\mathrm{MR}S_{\alpha}$ are identified correspondingly.

In terms of the independent validation of $\mathrm{MR}S_{\alpha}$, we calculate $\mathrm{MR}S_{\alpha}$ using the identified associated taxa in the independent validation cohort and perform the ROC analysis. If the independent validation cohort is limited, we employ CV to perform independent internal validation.

In this manuscript, CV is employed to perform independent internal validation in the NYULH COVID-19 and TEDDY studies, due to the lack of independent additional samples. For the GMHI multi-study cohort, we perform an independent evaluation using its independent external samples.

In addition, in the NYULH COVID-19 studies, the $\mathrm{MR}S_{\alpha}$s for the metagenomic and metatranscriptomic data are constructed similarly using the above analytic procedure above. In order to differentiate them in the Results section, we denoted the $\mathrm{MR}S_{\alpha}$ using the metagenomics and metatranscriptomic data by DNA_$\mathrm{MR}S_{\alpha}$ and RNA_$\mathrm{MR}S_{\alpha}$, respectively.

## Host risk score calculation

For the transcriptomic data in the NYULH COVID-19 study, we employed DESeq2 [2] to evaluate the association effects of the genes on the deceased/alive status. DESeq2 is a popular method to test for differential gene expression and it assumes that the normalized data follow the negative binomial distribution . We employed the DESeq function from the DESeq2 package with default settings to analyze host transcriptomic data. The function consists of three steps: (1) estimation of size factors, which are used to normalize library sizes in a model-based fashion; (2) estimation of dispersions from the negative binomial likelihood for each feature, and subsequent shrinkage of each dispersion estimate towards the parametric (default) trendline by empirical Bayes; (3) fitting each feature to the specified class groupings with negative binomial generalized linear models and performing hypothesis testing, for which the default Wald test is used. DESeq2 outputs the logarithmic fold change estimate (log2(case/control)) and p-value for each gene, respectively.

Due to the lack of independent validation samples in the NYULH COVID-19 study, CV was employed to perform the independent internal validation. Suppose the training dataset has $n$ subjects, $D$ genes and subscripts $i$, and $j$ indicate a subject and a gene, respectively. Let $G_{ij}$ be the read count, $i=1,\ldots,n$, and $j=1,\ldots,D$. We perform DESeq2 to test whether genes are differentially abundant between cases and controls and get the logarithmic fold change estimate $L_{j}$ and p-value $p_{j}$ for the $j$th gene, respectively.

With the outputs provided by DESeq2, the genes with p-values less than a given cut-off are selected for construction of transcriptomic risk score Host. For the $k$th p-value threshold, $D^{k}$ genes are selected and their read counts are $G_{ij_{k}}$, $j_{k}=1,\ldots,D^{k}$, $k=1,\ldots,K$. For $i$th sample, $\mathrm{Host}$ is calculated as

$\mathrm{Hos}t_{j_{k}}^{i}=\sum_{j=1}^{D^{k}} w_{j_{k}}\ln(G_{ij_{k}})$,

where $w_{j_{k}}=\mathrm{sign}(L_{j_{k}})$, $i=1,\ldots,n$. Similar to the procedure used in MRS construction, the P+T method and AUC evaluation are used to determine the optimal p-value cutoff and identify the candidate genes.

## Computation details

In this manuscript, 374 taxa in metagenome, 1, 149 taxa in metatranscriptome, and 14, 697 genes in host transcriptome data were analyzed in terms of DNA_$\mathrm{MR}S_{\alpha}$, RNA_$\mathrm{MR}S_{\alpha}$, and Host, respectively. The p-value cut-offs were 0.415, 0.02, and 0.00095 to identify the taxa/genes for the constructions of DNA_$\mathrm{MR}S_{\alpha}$, RNA_$\mathrm{MR}S_{\alpha}$, and Host, respectively. All identified features were reported in Table S6. The feature importance was determined by the selection proportion among all CV iterations.

**Reference**

1. Lin H, Peddada SD: **Analysis of compositions of microbiomes with bias correction**. *Nature communications* 2020, **11**(1):1-11.

2. Love MI, Huber W, Anders S: **Moderated estimation of fold change and dispersion for RNA-seq data with DESeq2**. *Genome biology* 2014, **15**(12):1-21.
